# Adherence to 24-hour movement guidelines in adolescence and its association with lower risk of hypertension in adulthood

**DOI:** 10.1007/s12519-025-00880-z

**Published:** 2025-03-06

**Authors:** Antonio García-Hermoso, José Francisco López-Gil, Rodrigo Yáñez-Sepúlveda, Jorge Olivares-Arancibia, Jacqueline Páez-Herrera, Yasmin Ezzatvar

**Affiliations:** 1 Navarrabiomed, Hospital Universitario de Navarra (HUN), Universidad Pública de Navarra (UPNA), IdiSNA, Pamplona, Spain; 2 One Health Research Group, Universidad de Las Américas, Quito, Ecuador; 3 Faculty Education and Social Sciences, Universidad Andrés Bello, Viña del Mar, Chile; 4 Grupo AFySE, Investigación en Actividad Física y Salud Escolar, Escuela de Pedagogía en Educación Física, Facultad de Educación, Universidad de Las Américas, Santiago, Chile; 5 Grupo Investigación Efidac, Escuela Educación Física, Pontificia Universidad Católica de Valparaíso, Valparaíso, Chile; 6 Lifestyle Factors With Impact On Ageing and Overall Health (LAH) Research Group, Department of Nursing, University of València, Valencia, Spain

**Keywords:** Blood pressure, Physical activity, Screen time, Sleep duration, Vascular health

## Abstract

**Background:**

There is limited research on how adherence to 24 h movement guidelines from adolescence to adulthood affects long-term hypertension outcomes. This study examined the association between sustained adherence to these guidelines and hypertension risk.

**Methods:**

Analysis was done on data from adolescents 12- to 19-year-olds who took part in Waves I and V of the Add Health Study. Physical activity (PA), screen time, and sleep duration were assessed through self-report questionnaires. Blood pressure (BP) was assessed on the right arm following a 5 min seated rest, utilizing an oscillometric device, and hypertension was defined as systolic/diastolic BP ≥ 140/90 mmHg, physician-diagnosed hypertension, or current antihypertensive medication use.

**Results:**

This prospective study included a total of 3076 participants (60.3% female), and 802 were diagnosed with hypertension. Meeting sleep duration guidelines at Wave I was associated with reductions in systolic [− 0.568 mmHg, 95% bias-corrected and accelerated (BCa) confident interval (CI) − 2.128 to − 0.011, *P* = 0.044] and diastolic (− 0.331 mmHg, 95% BCa CI − 1.506 to − 0.071, *P* = 0.043) BP at Wave V. Adherence to PA and sleep duration guidelines at both waves further reduced BP, with the greatest decreases observed among participants meeting all three guidelines: systolic (− 6.184 mmHg, 95% BCa CI − 13.45 to − 0.915, *P* = 0.040) and diastolic BP (− 3.156 mmHg, 95% BCa CI − 6.413 to − 0.120, *P* = 0.047). The risk of hypertension was lower among those who met the PA guidelines individually [relative risk (RR) 0.710, 95% CI 0.516–0.976, *P* = 0.035] or adhered to all three recommendations (RR 0.699, 95% CI 0.311–0.899, *P* = 0.030) in both waves.

**Conclusions:**

Our findings highlight the cardiovascular benefits of consistently adhering to healthy movement behaviors from adolescence through adulthood.

**Graphical abstract:**

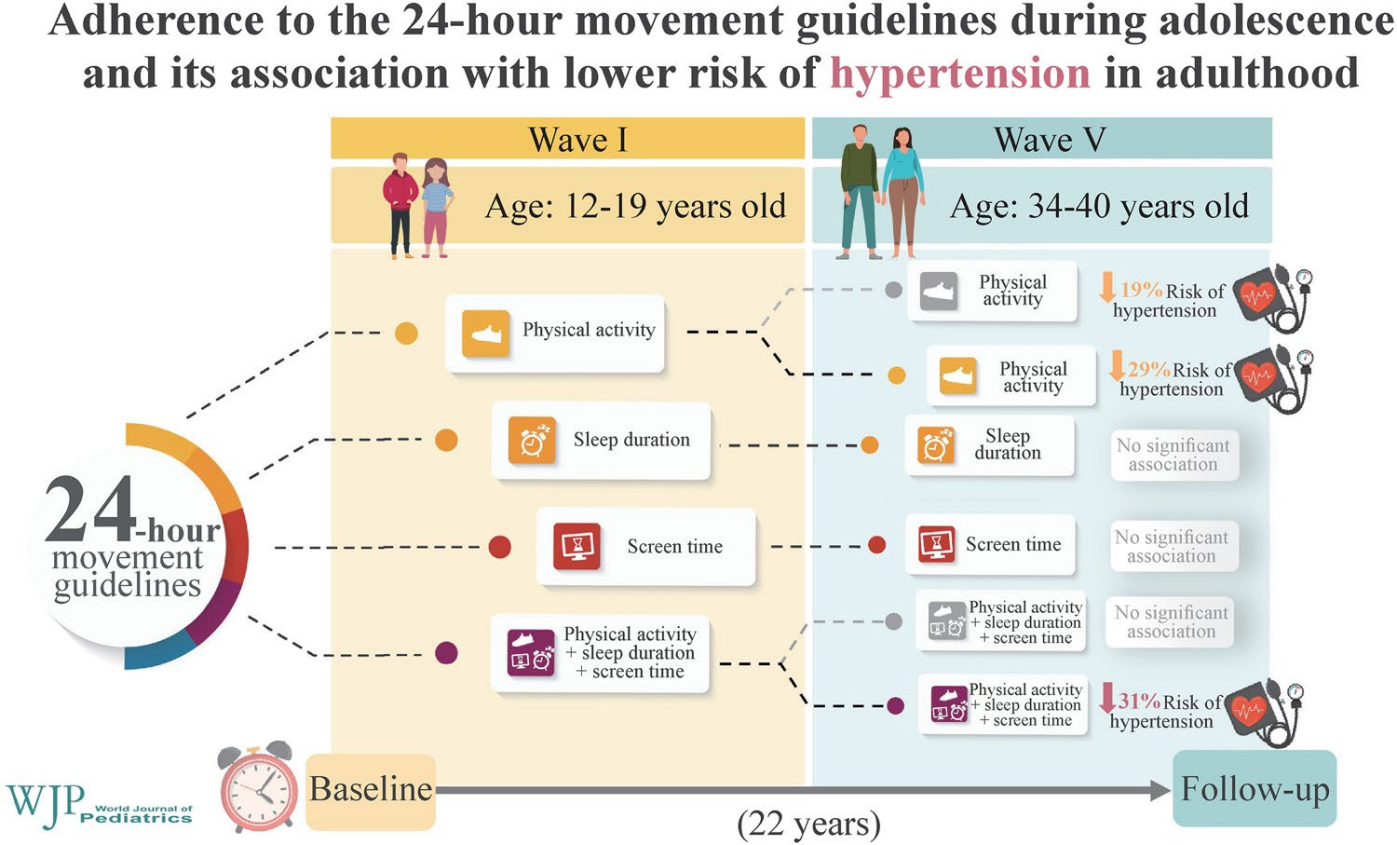

## Introduction

Globally, hypertension persists as a significant contributor to mortality, responsible for 10.4 million deaths and plays a prominent role in driving cardiovascular disease complications and fatalities [1, 2]. While various lifestyle factors, such as diet, sleep patterns, and physical activity (PA), have been extensively studied for their roles in hypertension prevention and management, emerging evidence highlights the importance of considering a holistic approach to daily movement behaviors [3, 4]. The 24 hour movement guidelines propose an integrated framework that includes PA, sedentary behavior, and sleep duration, emphasizing that health benefits arise from the balanced combination of these behaviors throughout an entire day [5, 6].

Adolescence represents a pivotal period for developing lifelong health behaviors, and maintaining healthy movement patterns during this time can have profound implications for health in adulthood [7]. Previous studies have reported that adherence to 24 hour movement guidelines during early life promotes both physical and mental benefits in adulthood, including lower rates of obesity [8], type 2 diabetes [9], and even lower midlife mortality [10]. However, there is limited research on how adherence to 24 hour movement guidelines from adolescence to adulthood affects long-term hypertension outcomes. Notably, a cross-sectional study of Chilean adults revealed that failing to meet any of the 24 hour movement guidelines was linked to a greater likelihood of hypertension [11]. Although the ideal regimen for vascular health is still under investigation, existing evidence indicates that maintaining physical activity and optimizing both the quantity and quality of sleep are advantageous for vascular health in older adults [4]. This highlights the importance of having balanced daily movement behavior to mitigate the risk of hypertension and to promote overall cardiovascular health.

The objective of the present study was to analyze the association between adherence to 24 hour movement guidelines in adolescence and the risk of hypertension in adulthood. We hypothesize that individuals who consistently meet PA guidelines, sedentary behavior, and sleep in adolescence and in adulthood will have a lower risk of hypertension than those who do not.

## Methods

### Participants

**Fig. 1:**
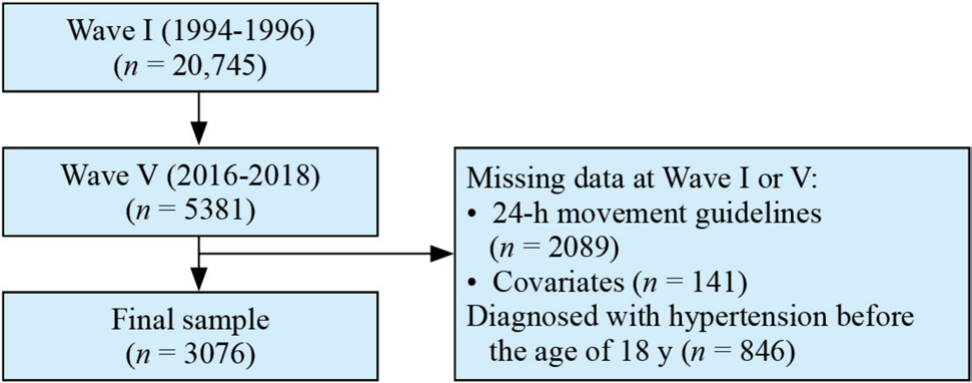
Flow chart of the study

For this longitudinal study, data were taken from the Add Health project, which followed a nationally representative group of U.S. adolescents in grades 7–12 into adulthood. Wave I, carried out in 1994, involved 20,745 in-home interviews [12]. Data from Wave V and biomarker data from 2016 to 2018 (*n* = 5381, age 33–39 years) were also used. After missing data were excluded and individuals who were diagnosed with hypertension before the age of 18 were removed, 3076 individuals were included in the analysis (Fig. 1). Among participants included and excluded in Wave V, there were no statistically significant differences in screen time (*P* = 0.514), sleep duration (*P* = 0.701), PA (*P* = 0.762), or demographic characteristics (ethnicity, *P* = 0.333; region, *P* = 0.324). Consequently, it is unlikely that the missing data significantly influenced the analysis results.

### Anthropometry

Weight and height were recorded during Wave V, and the formula used to calculate body mass index (BMI) was weight (kg) divided by height (m) squared.

### Blood pressure

A calibrated oscillometric blood pressure (BP) instrument (BP3MC1-PC-IB, Micro-Life USA; Dunedin, Finland) with a suitable cuff was used by skilled professional field workers to measure BP. After five minutes of sat rest, three systolic and diastolic BP readings were recorded on the right arm at 30-s intervals. The analysis was based on the average of the last two readings. The American Heart Association and American College of Cardiology’s 2017 guidelines [13] defined hypertension as a systolic blood pressure of 130 mmHg or higher, a diastolic BP of 80 mmHg or higher, a history of hypertension diagnosed by a doctor, or the use of antihypertensive drugs at the time of the diagnosis.

### Twenty-four-hour movement behavior

Detailed information on how PA, screen time, and sleep duration described elsewhere in a related publication [14].

#### Physical activity

Adolescents and adults reported their moderate-to-vigorous physical activity (MVPA) over the past week during the Wave I and Wave V in-home interviews, using specific questions and a scale detailed in the article referenced above [14]. Individuals meeting the PA guidelines were those who reported engaging in MVPA five or more times weekly, based on the Gordon-Larsen et al. criterion [15].

#### Screen time

Screen time was assessed through questions about weekly hours spent watching TV, videos, and playing video/computer games [14]. Total weekly screen time was calculated by summing responses. Adherence to guidelines was defined as ≤ 2 h/day for adolescents in Wave I and ≤ 3 h/day for adults in Wave V [16].

#### Sleep duration

During the Wave I and Wave V in-home interviews, sleep duration was self-reported by adolescents and adults. Adherence to sleep guidelines was evaluated based on National Sleep Foundation recommendations: 9–11 hours for ages 12–13, 8–10 hours for ages 14–17 [17], and 7–9 hours for adults in Wave V [16].

#### Trajectories

We analyzed the individuals who maintained each movement behavior individually in both waves and performed a general analysis in which we evaluated whether the individuals met all three guidelines in both waves.

### Definitions of covariates

Sociodemographic data, including age, sex, and ethnicity, were collected via in-home questionnaires.

Alcohol consumption was measured by asking participants how many days they had consumed alcohol (beer, wine, liquor) in the past 30 days. Responses were used to classify participants into categories ranging from “None” to “Every day or almost every day,” with intermediate groups reflecting varying frequencies over the past year.

Finally, smoking behavior was assessed by asking adults about cigarette use in the past 30 days, while fast food consumption was evaluated based on the number of days participants ate at fast-food establishments in the past week.

### Statistical analysis

The analytic sample, including tests for significant changes based on BP state, was first described. For continuous variables, descriptive statistics are displayed as means and standard deviations; for categorical variables, they are displayed as counts and percentages. Every model assumption, including homoscedasticity and normalcy, was confirmed.

For each of the 24 h movement patterns at Wave I, we compared the BP characteristics of those who did and did not meet the movement recommendations at Wave V. Furthermore, we compared the BP characteristics of those who did not met the guidelines with those who did at both time points (i.e., Waves I and V). After controlling for sex, ethnicity, age at Wave V, BMI at Wave V, alcohol consumption at Wave V, smoking habits at Wave V, and fast-food consumption at Wave V, generalized linear models with Gaussian distributions were used to analyze the main effects and interactions and control for potential confounding variables. A nonparametric approach that involved resampling dependent variables with replacement and bootstrapping with 5000 repeats was used.

Finally, using the previously indicated adjustments and variables, generalized linear models with binomial distributions were used to calculate the probabilities of developing hypertension in relation to fulfilling the 24 h movement criteria (individually and collectively). For the analysis, we used version 17.0 of STATA statistical software (StataCorp LLC, College Station, TX). The threshold for statistical significance was set at *P* < 0.05.

## Results

The average age of the participants during adolescence in Wave I was 15.30 years, whereas in adulthood, it was 37.65 years. Table 1 displays the general characteristics of the study sample by hypertension status at Wave V. Significant differences were observed between individuals with hypertension and those without hypertension in terms of age, sex, BMI, race/ethnicity, and smoking habits (*P* < 0.001). However, alcohol consumption did not significantly vary between groups (*P* = 0.748). Only 40 subjects met all three guidelines at both time points, with 33 individuals without hypertension and 7 with hypertension (*P* = 0.031).

**Table 1:** Demographic characteristics of the participating subjects by hypertension status at Wave V

Variables	Nonhypertension *n* = 2274	Hypertension *n* = 802	*P*
Age, y	37.57 (1.60)	37.87 (1.58)	< 0.001
Female sex, *n* (%)	1470 (64.6)	385 (48.0)	< 0.001
Body mass index, kg/m^2^	29.02 (7.23)	33.86 (8.19)	< 0.001
Race/ethnicity, *n* (%)			< 0.001
White	1731 (76.8)	560 (70.5)	
Black or African American	365 (16.2)	189 (23.8)	
American Indian or Alaska Native	29 (1.3)	10 (1.3)	
Asian	130 (5.8)	35 (4.4)	
Alcohol consumption, *n* (%)			0.748
None	145 (6.7)	62 (8.2)	
1–2 d in the past 12 mon	262 (12.1)	105 (13.9)	
Once a month or less (3 to 12 times in the past 12 mon)	478 (22.1)	121 (16.0)	
2–3 d/month	421 (19.5)	155 (20.6)	
1–2 d/wk	482 (22.3)	158 (21.0)	
3–5 d/wk	278 (12.9)	96 (12.7)	
Every day or almost every day	93 (4.3)	57 (7.6)	
Cigarette use in the past 30 d	4.73 (10.46)	6.36 (11.58)	< 0.001
24-h movement guidelines			
All three guidelines at Wave I, *n* (%)	195 (7.5)	62 (6.3)	0.202
All three guidelines at Wave V, *n* (%)	578 (25.4)	174 (21.7)	0.035
Meet at both moments, *n* (%)	33 (1.5)	7 (0.9)	0.031
Fast-food consumption last week	1.74 (2.13)	2.27 (2.46)	< 0.001

Table 2 illustrates the significant mean differences in systolic and diastolic BP at Wave V based on adherence to 24 h movement guidelines. At Wave I, meeting the sleep duration guidelines was associated with a significant mean reduction in systolic BP of − 0.568 mmHg [95% bias-corrected and accelerated (BCa) confidence interval (CI) − 2.128 to − 0.011, *P* = 0.044] and a decrease in diastolic BP of − 0.331 mmHg (95% BCa CI − 1.506 to − 0.071, *P* = 0.043). Adherence to PA or screen time guidelines alone did not yield significant changes in BP. When adherence to guidelines at both Wave I and Wave V was examined, significant reductions in BP were observed for the PA and sleep duration guidelines. Meeting the PA guidelines at both waves was linked to a mean decrease of − 0.912 mmHg in systolic BP (95% BCa CI − 2.111 to − 1.101, *P* = 0.020) and − 1.121 mmHg in diastolic BP (95% BCa CI − 2.143 to − 0.094, *P* = 0.049). Similarly, adherence to the sleep duration guidelines at both waves led to a decrease of − 1.173 mmHg in systolic BP (95% BCa CI − 2.114 to − 0.239, *P* = 0.037). Importantly, meeting all three movement behavior guidelines at both Wave I and Wave V resulted in the largest reductions: − 6.184 mmHg in systolic BP (95% BCa CI − 13.45 to − 0.915, *P* = 0.040) and − 3.156 mmHg in diastolic BP (95% BCa CI − 6.413 to − 0.120, *P* = 0.047).

**Table 2:** Mean differences in blood pressure at Wave V among those who did and did not meet guidelines for each of the 24-hour movement behaviors

Categories	Systolic blood pressure (mmHg)			Diastolic blood pressure (mmHg)		
	MD	95% BCa CI	*P*	MD	95% BCa CI	*P*
24-h movement guidelines at Wave I^a^						
Physical activity guideline	− 0.605	− 2.401 to 1.191	0.508	− 0.484	− 1.837 to 0.869	0.483
Screen time guideline	− 0.090	− 1.670 to 1.489	0.911	− 0.563	− 1.753 to 0.626	0.353
Sleep duration guideline	** − 0.568**	** − 2.128 to − 0.011**	**0.044**	** − 0.331**	** − 1.506 to − 0.071**	**0.043**
All-three guidelines	− 0.141	− 2.111 to 0.911	0.312	− 0.531	− 3.005 to 0.1942	0.673
24-h movement guidelines at Wave I and V^b^						
Physical activity guideline	** − 0.912**	** − 2.111 to − 1.101**	**0.020**	** − 1.121**	** − 2.143 to − 0.094**	**0.049**
Screen time guideline	− 0.441	− 1.165 to 0.525	0.413	− 0.900	− 1.612 to 0.114	0.094
Sleep duration guideline	** − 1.173**	** − 2.114 to − 0.239**	**0.037**	− 0.632	− 1.543 to 0.290	0.199
All-three guidelines	** − 6.184**	** − 13.45 to − 0.915**	**0.040**	** − 3.156**	** − 6.413 to − 0.120**	**0.047**

Analyses were adjusted for sex, race/ethnicity, age at follow-up, body mass index at follow-up, alcohol consumption at follow-up, smoking habit at follow-up, and fast-food consumption at follow-up

^a^Reference: nonmeeting the guidelines

^b^Reference: nonmeeting the guidelines at either time point (Wave I or V)

Values in bold indicate statistical significance (*P* < 0.05)

*BCa* Bias-corrected and accelerated, *MD* mean difference, *CI* confidence interval

Finally, Table 3 presents the relative risks (RRs) for hypertension at Wave V on the basis of adherence to 24 h movement behavior guidelines. In wave I, adherence to the PA guidelines was significantly associated with a reduced risk of hypertension, with an RR of 0.801 (95% CI 0.716 to 0.918, *P* = 0.020). However, no significant associations were observed for screen time, sleep duration, or adherence to all three guidelines at this time point. In contrast, adherence to guidelines at both Wave I and Wave V revealed stronger and more consistent reductions in hypertension risk. Participants meeting the PA guidelines across both waves had a significantly reduced RR of 0.710 (95% CI 0.516 to 0.976, *P* = 0.035). The most substantial benefit was observed among those meeting all three guidelines at both time points, with an RR of 0.699 (95% CI 0.311 to 0.899, *P* = 0.030).

**Table 3:** Relative risks for hypertension at Wave V among those who did and did not meet guidelines for each of the 24-h movement behaviors during adolescence and adulthood

Categories	RR	95% CI	*P*
24-hour movement guidelines at Wave I^a^			
Physical activity guideline	**0.801**	**0.716 to 0.918**	**0.020**
Screen time guideline	0.925	0.691 to 1.239	0.603
Sleep duration guideline	0.982	0.738 to 1.308	0.901
All-three guidelines	0.831	0.455 to 1.053	0.076
24-h movement guidelines at Wave I and V^b^			
Physical activity guideline	**0.710**	**0.516 to 0.976**	**0.035**
Screen time guideline	0.981	0.704 to 1.366	0.909
Sleep duration guideline	0.847	0.582 to 1.232	0.386
All-three guidelines	**0.699**	**0.311 to 0.899**	**0.030**

Analyses were adjusted for sex, race/ethnicity, age at follow-up, body mass index at follow-up, alcohol consumption at follow-up, smoking habit at follow-up, and fast-food consumption at follow-up

^a^Reference (RR = 1.00): nonmeeting the guideline

^b^Reference (RR = 1.00): nonmeeting the recommendation at either time point (Wave I or V)

Values in bold indicate statistical significance (*P* < 0.05)

*RR* relative risk, *CI* confidence interval

## Discussion

Our study highlights the significant association between adhering to 24-hour movement guidelines and BP levels, as well as the risk of hypertension, from adolescence into adulthood. The results particularly highlight the importance of consistently maintaining all three behaviors—PA, sleep duration, and limited screen time—across both waves, from adolescence to adulthood.

First, our findings align with existing evidence that identifies adequate sleep as a crucial factor in cardiovascular health [18]. The amount of sleep has an impact on BP management through a number of mechanisms, such as the control of stress hormones like cortisol and the alteration of autonomic nervous system activity [19]. Adequate sleep also helps maintain a balanced circadian rhythm, which is essential for BP homeostasis [20].

Regular PA from baseline and across both waves is associated with reduced BP, emphasizing the importance of maintaining an active lifestyle over the years. PA has long been recognized for its role in cardiovascular health, improving endothelial function, reducing arterial stiffness, and enhancing overall vascular function [21]. Regular PA contributes to lower BP by increasing nitric oxide availability [22], which promotes vasodilation, and by reducing the activity of the sympathetic nervous branch, which reduces vascular resistance [23]. When combined with adequate sleep, the synergistic effects of these behaviors further amplify their positive impact on BP regulation [24].

Moreover, adherence to all three guidelines (PA, sedentary behavior, and sleep duration) across both waves led to substantial decreases in both systolic and diastolic BP, as well as a lower RR for hypertension. These findings emphasize the importance of a comprehensive approach to daily movement recommendations for long-term cardiovascular health [5, 6]. The 24 hour movement guidelines highlight the integration of PA, limited sedentary behavior, and adequate sleep, recognizing that each component plays a critical role in maintaining optimal cardiovascular function [25]. By adhering to these guidelines from adolescence into adulthood, individuals can significantly mitigate the likelihood of developing hypertension later in life, confirming the findings from a study on Chilean adults, which showed that, compared with those who met all the guidelines, not meeting any was associated with greater odds of hypertension [11].

There are several limitations to acknowledge. First, the longitudinal study design prevents us from establishing causal-effect relationships. Second, participants may have overestimated or underestimated their behavior because information about 24 hour movement guidelines was self-reported. Self-reported measures may be biased, and PA questions lacked details on duration or typicality. Assumptions were made about activity intensity and duration, using “5 or more MVPA sessions per week” as a proxy for meeting PA guidelines. Fourth, we used data from 1994 to 1996 to gauge adherence to the guidelines established later. Over the past 20 years, there has been a substantial shift in how people use screens (from traditional television content to streaming), which has resulted in an increase in screen time. Therefore, the study’s findings should be interpreted with caution. Fifth, while the prescription dose for PA is clear, the recommended prescriptions for the other movement behaviors (screen time and sleep) are much vaguer [25]. This lack of clarity can complicate efforts to precisely gauge and prescribe balanced movement behaviors for optimal cardiovascular health. Sixth, there are no data on BP outcomes during adolescence, so we could not adjust the analysis for baseline levels. However, we excluded adolescents who were diagnosed with hypertension before 19 years of age (*n* = 73). Seventh, one minor limitation is the generalizability of the findings, as the cohort may not represent the full diversity of populations. Another limitation is the lack of information on whether any participants had sleep disorders, which could have significantly influenced the results. Sleep disorders such as insomnia or sleep apnea can affect both sleep quality and BP [26], potentially confounding the relationship between adherence to sleep guidelines and hypertension risk.

In conclusion, our study revealed that adhering to 24 hour movement guidelines significantly reduces BP and hypertension risk over time, especially when these behaviors are maintained consistently at both waves. Educational programs and interventions should focus on encouraging regular PA, reducing screen time, and ensuring adequate sleep among adolescents to foster lifelong vascular health. Further studies should investigate the specific factors through which these behaviors interact and contribute to BP regulation and identify strategies to enhance adherence to these guidelines in various populations.

## Data Availability

Due to our data protection agreements with the participating cohort study, we are unable to share individual-level data with third parties. According to Add Health’s data access policy, researchers can submit data requests to the steering committee. These requests will be reviewed promptly for confidentiality, data protection, and intellectual property considerations, and will not be unreasonably denied. Researchers registered with Add Health can apply for access to its database by submitting an application (https://data.cpc.unc.edu/projects/2/view).
